# Immunohistochemical analysis of organic anion transporter 2 and reduced folate carrier 1 in colorectal cancer: Significance as a predictor of response to oral uracil/ftorafur plus leucovorin chemotherapy

**DOI:** 10.3892/mco.2013.104

**Published:** 2013-04-15

**Authors:** SATOSHI NISHINO, AYUMI ITOH, HIROSHI MATSUOKA, KOTARO MAEDA, SHINGO KAMOSHIDA

**Affiliations:** 1 Laboratory of Pathology, Department of Medical Biophysics, Kobe University Graduate School of Health Sciences, Kobe, Hyogo 654-0142;; 2 Department of Surgery, School of Medicine, Fujita Health University, Toyoake, Aichi 470-1192, Japan

**Keywords:** organic anion transporter 2, reduced folate carrier 1, uracil/ftorafur, leucovorin, colorectal cancer

## Abstract

Colorectal cancer is one of the most common malignancies in developed countries and chemotherapy is the standard treatment option for advanced colorectal cancer. Identification of biomarkers for predicting response to uracil/ftorafur plus leucovorin (UFT/LV) chemotherapy is an important issue in colorectal cancer treatment. Organic anion transporter 2 (OAT2) and reduced folate carrier 1 (RFC1) are the major uptake transporters of 5-fluorouracil (5-FU) and LV, respectively. In the present study, the correlation between OAT2 and RFC1 expression and histological response to preoperative UFT-based (UFT or UFT/LV) chemotherapy was investigated. Pre-treatment biopsy specimens obtained from 45 patients were evaluated for OAT2 and RFC1 expression levels by using an immunohistochemical approach. A high expression of OAT2 and RFC1 was significantly correlated with good response to UFT-based chemotherapy (P<0.0001 and P= 0.002, respectively). In multivariate logistic regression analysis, a high OAT2 expression was an independent predictor of good response to UFT-based chemotherapy (P=0.02), unlike RFC1 expression. High expression levels of OAT2 were significantly correlated with a good response in the UFT-treated (P= 0.04) as well as the UFT/LV-treated (P<0.0005) groups; however, RFC1 expression levels were significantly correlated with a good response only in the UFT/LV-treated group (P=0.02). Therefore, immunohistochemical analysis of OAT2 and RFC1 may serve as a useful tool for predicting the efficacy of UFT/LV treatment regimens in colorectal cancer patients.

## Introduction

Colorectal cancer is one of the most common malignancies in developed countries. Chemotherapy is the standard treatment option for advanced colorectal cancer. Uracil plus ftorafur (UFT) is an oral anticancer drug composed of 1-(tetrahydro-2-furanyl)-5-fluorouracil (ftorafur) and uracil, at a molar ratio of 1:4 (1,2). Ftorafur, a prodrug of 5-fluorouracil (5-FU), is converted to 5-FU by hepatic metabolism following gastrointestinal absorption (3). Uracil competitively inhibits the degradation of 5-FU by dihydropyrimidine dehydrogenase. Thus, UFT increases 5-FU concentration within the tumor site. Leucovorin (5-formyltetrahydrofolate; LV) itself possesses no antitumor activity; however, it enhances the anticancer activity of 5-FU by providing a stable supply of 5,10-methylenetetrahydrofolate (reduced folate; CH_2_THF) (4). UFT plus LV (UFT/LV) is widely accepted as a chemotherapeutic regimen for advanced colorectal cancer, due to its comparable efficacy to intravenous 5-FU plus LV (5-FU/LV), its more favorable toxicity profile and its convenience (by eliminating the need for repeated intravenous injections of 5-FU) (5–8). Although 5-FU/LV plus oxaliplatin (FOLFOX) and 5-FU/LV plus irinotecan (FOLFIRI) have been widely used, UFT/LV chemotherapy remains the backbone of colorectal cancer treatment. Therefore, the identification of chemosensitivity markers that may predict response to UFT/LV chemotherapy is a useful approach to the individualization of colorectal cancer treatment and it may help avoid the administration of inappropriate chemotherapeutic regimens with unpleasant side effects.

The effectiveness of chemotherapy is dependent on the intracellular accumulation of the anticancer drugs, which may be altered by uptake and efflux transporters. Previously, the majority of investigations on drug transporters has focused on the drug efflux transporters and their ability to confer multidrug resistance. However, mechanisms of uptake into the tumor cells may prove even more important compared to efflux mechanisms in predicting the efficacy of anticancer drugs, since they determine intracellular drug concentrations (9,10). The organic anion transporter (OAT) family of proteins are essential for the uptake of endogenous compounds, a variety of xenobiotics and clinically important drugs (11,12). OAT2, also known as SLC22A7, mediates the sodium-independent uptake of anticancer drugs, including 5-fluorouracil, methotrexate and paclitaxel (13).

Reduced folate carrier 1 (RFC1), also referred to as SLC19A1, is the major transporter of folates and methotrexate in mammalian cells (14). LV is preferred to folate as it is already reduced and may therefore enter the cytoplasm via RFC1 (15). It is suggested that the expression of OAT2 and RFC1 in tumor cells may be of predictive value for the effectiveness of UFT/LV chemotherapy in colorectal cancer patients. However, the role of the drug uptake transporters in UFT/LV chemotherapy has not been elucidated. In the present study, we used an immunohistochemical approach to investigate the correlation between OAT2 and RFC1 expression and histological response to preoperative UFT-based (UFT or UFT/LV) chemotherapy in colorectal cancer, with the aim of identifying predictive biomarkers for the efficacy of UFT/LV chemotherapy.

## Materials and methods

### Patients and specimens

The study population included 45 colorectal cancer patients (28 male and 17 female), with a median age of 60 years (range, 33–81 years). The patients had received preoperative chemotherapy for 2 weeks, until 1 day prior to surgical resection at the Fujita Health University Hospital, Aichi, Japan, between 2001 and 2009. The chemotherapeutic regimen was UFT (450–600 mg/body/day) for 24 patients and UFT (450–600 mg/body/day) plus LV (75 mg/body/day) for the remaining 21 patients. No other treatment was administered preoperatively. The clinicopathological characteristics of the patients are provided in Table I. Informed consent for the administration of preoperative chemotherapy as well as the use of tumor tissue for analyzing protein expression was obtained from all patients. This study was approved by the Ethics Committee of Kobe University Graduate School of Health Sciences and Fujita Health University School of Medicine.

Adequate biopsy material from ≥2 cancerous sections was obtained from all patients prior to administration of preoperative chemotherapy. The pre-treatment biopsies and post-treatment resection specimens were routinely fixed in 10% formalin and embedded in paraffin wax. Sections (3 *μ*m) were cut and mounted on aminopropyltriethoxysilane slides, then stained with hematoxylin and eosin (H&E) to assess histopathological features and chemotherapeutic effects.

### Histological evaluation of chemotherapeutic effects

Histological response was evaluated by grading the post-treatment resection specimens according to the Japanese Classification of Colorectal Carcinoma (16). Major grading (grades 0–3) and additional minor grading for grade 1 (grades 1a and 1b) were classified as follows: grade 0, no change; grade 1a, necrosis or disappearance of the tumor in <1/3 of the whole lesion; grade 1b, necrosis or disappearance of the tumor in >1/3 but in <2/3 of the whole lesion; grade 2, necrosis or disappearance of the tumor in >2/3 of the whole lesion, with viable tumor cells; and grade 3, necrosis of the whole lesion and/or replacement by fibrotic tissue, with no viable tumor cells. The response of tumors with grades 1b and 2 was classified as ‘good histological response,’ and that of tumors with grades 0 and 1a as ‘poor histological response’, according to a previous study (17). No tumors with grade 3 response were identified in our study.

### Immunohistochemistry

Immunohistochemical staining of formalin-fixed, paraffin-embedded tumor sections was performed using a rabbit polyclonal anti-OAT2 antibody (dilution 1:100, TransGenic, Kumamoto, Japan) and a rabbit polyclonal anti-RFC antibody (dilution 1:400, Atlas Antibodies, Stockholm, Sweden). Sections were deparaffinized in xylene and rehydrated in graded alcohols. Endogenous peroxidase activity was blocked by treatment with 0.3% hydrogen peroxide in methanol for 30 min. For antigen retrieval, pressure cooking was performed for 10 min at 120°C in optimal soaking solutions: 0.001 mol/l ethylenediaminetetraacetic acid (EDTA) (pH 8.0) for OAT2 and 0.01 mol/l Tris base containing 0.001 mol/l EDTA (pH 9.0) for RFC1. After pressure cooking, the sections were cooled in the soaking solution at room temperature (RT) for 30 min. The sections were washed under running tap water, followed by 0.01 M phosphate-buffered saline (PBS, pH 7.2). After washing, the sections were incubated with the primary antibodies overnight at RT. The sections were then washed in PBS and incubated with Histofine Simple Stain MAX PO (Nichirei, Tokyo, Japan) for 1 h at RT. The reaction products were detected using a diaminobenzidine solution (Dako, Glostrup, Denmark). Subsequently, the sections were washed, counterstained with Mayer’s hematoxylin, dehydrated through graded alcohols and xylene, and coverslipped. Negative controls were set up by the omission of the primary antibodies. Positive controls were formalin-fixed, paraffin-embedded tissue sections of normal kidney and normal placenta for OAT2 and RFC1, respectively.

### Scoring of immunostained tissue

The stained sections were independently reviewed by two investigators (S.N. and S.K.), who were blinded to the clinicopathological characteristics of the patients. Staining was regarded as positive when the tumor cells exhibited cytoplasmic and/or membrane staining. Semi-quantitative assessment of OAT2 and RFC1 expression levels was scored according to the staining intensity and percentage of positive tumor cells. Briefly, the staining intensity was scored as follows: 0, no staining; 1, weakly positive; 2, moderately positive; and 3, strongly positive. The percentage of positive tumor cells was scored as follows: 0, no positive tumor cells; 1, <40% positive cells; 2, 40–70% positive cells; and 3, ≥71% positive cells. A composite score was obtained by calculating the sum of the two scores. Any differences in the scores were discussed between the two investigators and consolidated into a final score.

### Statistical analysis

Receiver operating characteristic (ROC) curve analysis was used for selecting the optimal cut-off score to determine the threshold for a high expression level. According to the cut-off score determined by the optimal sensitivity and specificity (maximum sum of sensitivity and specificity), scores of 0–5 represented a ‘low expression level’ and a score of 6 represented a ‘high expression level’.

The Fisher’s exact test was used to evaluate the correlation between OAT2 and RFC1 expression levels with patient age and gender, tumor location, histological grade, depth of invasion, lymph node metastasis and distant metastasis. The Fisher’s exact test was also used to determine the association of chemotherapeutic response with patient age and gender, tumor location, histological grade, lymph node metastasis, distant metastasis, chemotherapeutic regimen and OAT2 and RFC1 expression. The correlation of expression levels between OAT2 and RFC1 was analyzed using Pearson’s test.

Variables with a P-value <0.35 in the Fisher’s exact test were included in a logistic regression model for univariate and multivariate analyses to assess the predictive factors that may affect the efficacy of preoperative chemotherapy. Statistical analyses were performed using a free statistical software EZR (Easy R) on R commander version 2.13.0 (Saitama Medical Center, Jichi Medical University, Saitama, Japan). P<0.05 was considered to indicate a statistically significant difference.

## Results

### Immunohistochemical findings

**Figure 1:**
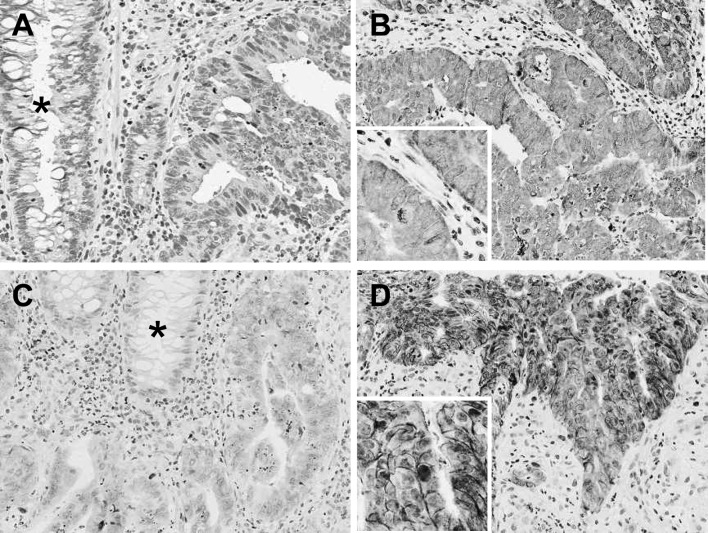
Representative patterns of immunostaining for organic anion transporter 2 (OAT2) and reduced folate carrier 1 (RFC1) in the pre-treatment biopsy specimens from colorectal cancer patients treated with preoperative uracil plus ftorafur (UFT)-based chemotherapy. (A) Low OAT2 expression in a case with poor (grade 0) histological response and (B) high OAT2 expression in a case with good (grade 2) histological response. (C) Low RFC1 expression in a case with poor (grade 0) histological response and (D) high RFC1 expression in a case with good (grade 2) histological response. Strong immunoreactivity was observed on the cell membrane/cytoplasm of numerous tumor cells (B and D, insets). However, no or weak expression of OAT2 and RFC1 was observed in the corresponding normal epithelia (A and C, asterisks).

OAT2 was relatively homogeneously distributed throughout the tumors; however, RFC1 exhibited heterogeneous distribution. Of the 45 pre-treated biopsy specimens, 18 (40%) exhibited high expression levels of OAT2 and 27 (60%) specimens exhibited a low OAT2 expression. Thirteen (29%) of the biopsy specimens exhibited high expression levels of RFC1 and 32 (71%) specimens exhibited low RFC1 expression. No significant correlation of expression levels was observed between OAT2 and RFC1 (r= 0.174; P= 0.252). Normal colorectal epithelia exhibited negative or weak staining for OAT2 and RFC1. The representative staining patterns of OAT2 and RFC1 are shown in Fig. 1.

### Correlation of expression levels of OAT2 and RFC1 with clinicopathological parameters

The correlation between expression levels of OAT2 and RFC1 in pre-treatment biopsy specimens and clinicopathological parameters is shown in Table I. High expression levels of OAT2 were observed in 10 (67%) of the 15 colon cancer patients and in 8 (27%) of the 30 rectal cancer patients (P=0.02). No significant association of OAT2 expression with the other clinicopathological parameters, including patient age and gender, histological grade, depth of invasion, lymph node metastasis, distant metastasis and chemotherapeutic regimen was observed. No significant correlation was observed between expression levels of RFC1 and any of the clinicopathological parameters.

### Correlation of clinicopathological parameters and expression levels of OAT2 and RFC1 with histological response to chemotherapy

Table II shows the correlation of clinicopathological parameters and OAT2 and RFC1 expression levels with histological response to preoperative chemotherapy. Good histological response was observed in 12 (27%) of the 45 tumors [5 (21%) of the 24 UFT-treated tumors and in 7 (33%) of the 21 UFT/LV-treated tumors]. No significant association was observed between histological response and clinicopathological parameters, including patient age and gender, tumor location, histological grade, depth of invasion, lymph node metastasis, distant metastasis and chemotherapeutic regimen.

A high OAT2 expression in the pre-treatment biopsies was significantly correlated with good histological response to UFT-based chemotherapy (P<0.0001): good histological response was observed in 11 (61%) of the 18 tumors exhibiting a high OAT2 expression and in 1 (4%) of the 27 tumors exhibiting a low OAT2 expression. In addition, a high RFC1 expression was correlated with good histological response (P= 0.002): good histological response was observed in 8 (62%) of the 13 tumors exhibiting high RFC1 expression and in 4 (13%) out of the 32 tumors exhibiting a low RFC1 expression.

Table III shows the results of the logistic regression analysis of predictive factors for histological response to UFT-based chemotherapy. OAT2 expression was identified as the most significant predictive factor for good histological response in the univariate analysis [odds ratio (OR): 0.03, 95% confidence interval (CI): 0.0006–0.24, P<0.0001], as well as in the multivariate analysis (OR: 106, 95% CI: 2.40–4650, P=0.02). RFC1 expression was identified as a predictive factor for good histological response in the univariate analysis (OR: 0.10, 95% CI: 0.02–0.51, P=0.002), but not in the multivariate analysis (OR: 117, 95% CI: 0.85–16100, P= 0.06). However, patient gender, histological grade, depth of invasion and lymph node metastasis were not identified as predictive factors for good histological response.

### Correlation of OAT2 and RFC1 expression with histological response according to chemotherapeutic regimen

As shown in Table IV, when separately analyzed according to the chemotherapeutic regimen, OAT2 expression levels were significantly correlated with good histological response in the UFT-treated and UFT/LV-treated groups. In the UFT-treated group, 4 (44%) of the 9 patients with high OAT2 expression levels and 1 (7%) of the 15 patients with low OAT2 expression levels exhibited a good histological response (P=0.04). In the UFT/LV-treated group, good histological response was observed in 7 (78%) of the 9 patients with high OAT2 expression levels and in none of the 12 patients with low OAT2 expression levels (P<0.0005: higher level of statistical significance compared to the UFT-treated group).

No association between RFC1 expression levels and histological response was observed in the UFT-treated group (P= 0.18). However, RFC1 expression levels correlated with good histological response in the UFT/LV-treated group. Good histological response was observed in 6 (67%) of the 9 patients with high RFC1 expression and in 1 (8%) of the 12 patients with low RFC1 expression (P=0.02). Six of the 7 patients in the UFT/LV-treated group who responded well, demonstrated high expression levels of both OAT2 and RFC1.

## Discussion

Oral UFT/LV chemotherapy remains the backbone of treatment for colorectal cancer; thus, the identification of chemosensitivity markers for predicting the response to UFT/LV chemotherapy may facilitate more effective and individualized treatment of this disease. Previously identified predictive chemosensitivity markers for UFT/LV chemotherapy in colorectal cancer include high mRNA expression levels of 5-FU metabolism-related enzymes, orotate phosphoribosyltransferase (18) and thymidine phosphorylase (19). However, although the expression of drug uptake transporters has been considered to be mechanistically and biologically associated with tumor chemosensitivity (9,10), the role of drug uptake transporters in colorectal cancer chemotherapy has not been elucidated. Since OAT2 and RFC1 are the major transporters of 5-FU and LV (13,15), respectively, the expression levels of OAT2 and RFC1 may be candidate biomarkers for the prediction of tumor response to UFT/LV chemotherapy. In the present study, we immunohistochemically investigated the correlation between expression levels of OAT2 and RFC1 and histological response to UFT-based chemotherapy.

High expression levels of OAT2 and RFC1 were observed in 40 and 29% of pre-treated colorectal cancer specimens, respectively, whereas the corresponding normal colorectal epithelia were negative or weakly positive for the two transporters. In accordance with our results, Seithel *et al* reported that *OAT2* mRNA is absent in the normal human colon (20). In addition, Odin *et al* reported that mean expression levels of the *RFC-1* gene were significantly higher in colorectal cancer tissues compared to the adjacent normal mucosa (21). The higher frequency of OAT2 and RFC1 expression in colorectal cancer specimens, compared to the corresponding normal tissues, may indicate that an increased expression of OAT2 and RFC1 is associated with the development of colorectal cancer.

We also demonstrated that high expression levels of OAT2 occurred significantly more frequently in colon cancer compared to rectal cancer (P= 0.02). Recently, Hlavata *et al* (22) reported that certain ATP-binding cassette (ABC) transporters exhibited significantly differential mRNA expression between colon and rectal cancer tissues. The mRNA levels of *ABCA12*, *ABCC7* and *ABCC8* increased in direction from the colon to the rectum, whereas *ABCB9*, *ABCB11*, *ABCG5* and *ABCG8* exhibited a significant reverse trend, i.e., a decrease in the levels in direction from the colon to the rectum. However, although we identified OAT2 to be differentially distributed between colon and rectal cancer tissues, this distribution pattern has not yet been investigated. Further investigations are required to elucidate the underlying mechanisms and biological significance of the differential transporter distribution.

In the univariate logistic regression analysis following adjustment for several clinical factors, high expression levels of OAT2 and RFC1 were identified as significant predictive factors of good histological response to UFT-based chemotherapy (P<0.0001 and P= 0.002, respectively). In the multivariate logistic regression analysis, the association between high expression levels of OAT2 and good response to UFT-based chemotherapy was also found to be significant, independent of the other clinicopathological factors (P=0.02). However, RFC1 expression was not confirmed as an independent predictive factor for tumor response to treatment.

When separately analyzed according to the chemotherapeutic regimen, high OAT2 expression levels were significantly correlated with good response in the UFT-treated (P=0.04) as well as in the UFT/LV-treated groups (P<0.0005: higher level of statistical significance compared to the UFT-treated group). However, high RFC1 expression was associated with good response in the UFT/LV-treated group (P=0.02) but not in the UFT-treated group. Furthermore, the majority of responders in the UFT/LV-treated group exhibited high expression levels of both OAT2 and RFC1. These results suggest that the predictive power of OAT2 and RFC1 expression was stronger for the UFT/LV combination therapy compared to the UFT monotherapy.

Based on the data of the present study, we hypothesized on the mechanism by which OAT2 and RFC1 expression levels are predictive of the response to UFT/LV chemotherapy. 5-FU is imported into the tumor cells via OAT2 and then metabolized to its active metabolite, 5-fluorodeoxyuridine monophosphate (FdUMP) (4). FdUMP binds to CH_2_THF and thymidylate synthase (TS) to form a ternary complex, resulting in the suppression of DNA synthesis through the inhibition of TS activity (4). High intracellular levels of CH_2_THF are required for optimal formation of the ternary complex. LV enters the cell via RFC1, increases the intracellular concentration of CH_2_THF and thus potentiates ternary complex formation (4). In patients with high OAT2 expression levels, large amounts of 5-FU are imported following UFT administration. In the UFT/LV-treated group, in patients with high expression levels of RFC1 as well as OAT2, increased LV uptake may lead to sufficient levels of CH_2_THF. Consequently, ternary complex formation and subsequent TS inhibition may be more efficient. However, in patients with a low RFC1 expression, CH_2_THF does not reach sufficient levels and the ternary complex may not be able to form as efficiently, resulting in poor TS inhibition, despite the administration of LV.

However, the contribution of OAT2 expression to the antitumor effect of UFT monotherapy was less significant compared to that of the UFT/LV combination therapy. Even if a sufficient amount of 5-FU enters the tumor cells via OAT2, the ternary complex may not be able to form sufficiently if the tumor cells are in a folate-deficient condition, resulting in poor TS inhibition. Furthermore, RFC1 expression levels may be irrelevant to the effect of UFT alone.

In conclusion, high expression levels of OAT2 and RFC1 in pre-treatment biopsy specimens were significantly correlated with the antitumor effect of UFT-based chemotherapy, particularly of UFT/LV regimens. Immunohistochemical analysis of OAT2 and RFC1 expression may be a useful tool to identify colorectal cancer patients who may benefit from treatment with an UFT/LV regimen. However, our findings were based on a retrospective analysis of a limited patient sample; thus, a large-scale prospective trial is required to confirm OAT2 and RFC1 expression as prognostic indicators in colorectal cancer patients receiving oral UFT/LV chemotherapy in the adjuvant setting.

## Figures and Tables

**Table I:** Correlation of OAT2 and RFC1 expression with clinicopathological parameters.

Parameters	OAT2 expression			RFC1 expression		
	High	Low	P-value	High	Low	P-value
Age (years)						
<60	6	16	0.13	6	16	1.00
≥60	12	11		7	16	
Gender						
Male	8	20	0.06	9	19	0.74
Female	10	7		4	13	
Tumor location						
Colon	10	5	0.02^a^	5	10	0.73
Rectum	8	22		8	22	
Histological grade						
Low (well-differentiated)	6	12	0.75	5	13	1.00
Intermediate (moderately differentiated)	11	15		8	18	
Depth of invasion						
Confined to the muscularis propria	6	6	0.50	3	9	1.00
Invading or exceeding the subserosa	12	21		10	23	
Lymph node metastasis						
Negative	8	12	1.00	9	11	0.05
Positive	10	15		4	21	
Distant metastasis						
Negative	18	25	0.51	13	30	1.00
Positive	0	2		0	2	
Chemotherapeutic regimen						
UFT	9	15	0.77	4	20	0.10
UFT/LV	9	12		9	12	

a Statistically significant. Histological grade was evaluated in 44 of the 45 tumors: 1 case of G3 (high grade, poorly differentiated) was excluded from the analysis. OAT2, organic anion transporter 2; RFC1, reduced folate carrier 1; UFT, uracil plus ftorafur; LV, leucovorin.

**Table II:** Correlation of clinicopathological parameters and OAT2 and RFC1 expression with histological response to UFT-based chemotherapy.

Variables	Good response	Poor response	P-value
Age (years)			
<60	5	17	0.74
≥60	7	16	
Gender			
Male	6	22	0.32
Female	6	11	
Tumor location			
Colon	4	11	1.00
Rectum	8	22	
Histological grade			
Low (well-differentiated)	3	15	0.30
Intermediate (moderately differentiated)	9	17	
Depth of invasion			
Confined to the muscularis propria	5	7	0.25
Invading or exceeding the subserosa	7	26	
Lymph node metastasis			
Negative	7	13	0.32
Positive	5	20	
Distant metastasis			
Negative	12	31	1.00
Positive	0	2	
Chemotherapeutic regimen			
UFT	5	19	0.50
UFT/LV	7	14	
OAT2 expression			
Low	1	26	<0.0001^a^
High	11	7	
RFC1 expression			
Low	4	28	0.002^a^
High	8	5	

a Statistically significant. Histological grade was evaluated in 44 of the 45 tumors: 1 case of G3 (high grade, poorly differentiated) was excluded from the analysis. OAT2, organic anion transporter 2; RFC1, reduced folate carrier 1; UFT, uracil plus ftorafur; LV, leucovorin.

**Table III:** Logistic regression analysis of predictive factors for histological response to UFT-based chemotherapy.

Variables	Univariate analysis		Multivariate analysis	
	OR (95% CI)	P-value	OR (95% CI)	P-value
Gender	0.51 (0.11–2.40)	0.32	7.70 (0.21–275)	0.26
Histological grade	0.39 (0.057–1.90)	0.30	9.50 (0.47–190)	0.14
Depth of invasion	2.60 (0.49–13)	0.25	0.03 (0.0005–2.3)	0.12
Lymph node metastasis	2.10 (0.46–10)	0.32	0.19 (0.009–4.0)	0.28
OAT2 expression	0.03 (0.0006–0.24)	<0.0001^a^	106 (2.40–4650)	0.02^a^
RFC1 expression	0.10 (0.02–0.51)	0.002^a^	117 (0.85–16100)	0.06

a Statisticaly significant. UFT, uracil plus ftorafur; OR, odds ratio; CI, confidence interval; OAT2, organic anion transporter 2; RFC1, reduced folate carrier 1.

**Table IV:** Correlation of OAT2 and RFC1 expression with histological response according to chemotherapeutic regimens.

Transporter expression	Good response	Poor response	P-value
UFT-treated group (n=25)			
OAT2-high	4	5	0.04^a^
OAT2-low	1	14	
RFC1-high	2	2	0.18
RFC1-low	3	17	
UFT/LV-treated group (n=20)			
OAT2-high	7	2	<0.0005^a^
OAT2-low	0	12	
RFC1-high	6	3	0.02^a^
RFC1-low	1	11	

a Statistically significant. OAT2, organic anion transporter 2; RFC1, reduced folate carrier 1; UFT, uracil plus ftorafur; LV, leucovorin.

## References

[1] Fujii S, Ikenaka K, Fukushima M, Shirasaka T (1978). Effect of uracil and its derivatives on antitumor activity of 5-fluorouracil and 1-(2-tetrahydrofuryl)-5-fluorouracil. Gann.

[2] Milano G, Ferrero JM, François E (2004). Comparative pharmacology of oral fluoropyrimidines: a focus on pharmacokinetics, pharmacodynamics and pharmacomodulation. Br J Cancer.

[3] Anttila MI, Sotaniemi EA, Kairaluoma MI, Mokka RE, Sundquist HT (1983). Pharmacokinetics of ftorafur after intravenous and oral administration. Cancer Chemother Pharmacol.

[4] Longley DB, Harkin DP, Johnston PG (2003). 5-Fluorouracil: mechanisms of action and clinical strategies. Nat Rev Cancer.

[5] Borner MM, Schöffski P, de Wit R (2002). Patient preference and pharmacokinetics of oral modulated UFT versus intravenous fluorouracil and leucovorin: a randomised crossover trial in advanced colorectal cancer. Eur J Cancer.

[6] Carmichael J, Popiela T, Radstone D (2002). Randomized comparative study of tegafur/uracil and oral leucovorin versus parenteral fluorouracil and leucovorin in patients with previously untreated metastatic colorectal cancer. J Clin Oncol.

[7] Douillard JY, Hoff PM, Skillings JR (2002). Multicenter phase III study of uracil/tegafur and oral leucovorin versus fluorouracil and leucovorin in patients with previously untreated metastatic colorectal cancer. J Clin Oncol.

[8] Lembersky BC, Wieand HS, Petrelli NJ (2006). Oral uracil and tegafur plus leucovorin compared with intravenous fluorouracil and leucovorin in stage II and III carcinoma of the colon: results from National Surgical Adjuvant Breast and Bowel Project Protocol C-06. J Clin Oncol.

[9] Dobson PD, Kell DB (2008). Carrier-mediated cellular uptake of pharmaceutical drugs: an exception or the rule?. Nat Rev Drug Discov.

[10] Sissung TM, Baum CE, Kirkland CT, Gao R, Gardner ER, Figg WD (2010). Pharmacogenetics of membrane transporters: an update on current approaches. Mol Biotechnol.

[11] Marzolini C, Tirona RG, Kim RB (2004). Pharmacogenomics of the OATP and OAT families. Pharmacogenomics.

[12] You G (2004). The role of organic ion transporters in drug disposition: an update. Curr Drug Metab.

[13] Kobayashi Y, Ohshiro N, Sakai R, Ohbatashi M, Kohyama N, Yamamoto T (2005). Transport mechanism and substrate specificity of human organic anion transporter 2 (hOat2 [SLC22A7]). J Pharm Pharmacol.

[14] Sirotnak FM, Tolner B (1999). Carrier-mediated membrane transport of folates in mammalian cells. Annu Rev Nutr.

[15] Blehaut H, Mircher C, Ravel A (2010). Effect of leucovorin (folinic acid) on the developmental quotient of children with Down’s syndrome (trisomy 21) and influence of thyroid status. PLoS One.

[16] Japanese Society for Cancer of the Colon and Rectum (2009). Japanese Classification of Colorectal Carcinoma.

[17] Kamoshida S, Matsuoka H, Shiogama K (2004). Immunohistochemical analysis of thymidylate synthase, p16^INK4a^, cyclin-dependent kinase 4 and cyclin D1 in colorectal cancers receiving preoperative chemotherapy: significance of p16^INK4a^-mediated cellular arrest as an indicator of chemosensitivity to 5-fluorouracil. Pathol Int.

[18] Ichikawa W, Uetake H, Shirota Y (2003). Both gene expression for orotate phosphoribosyltransferase and its ratio to dihydropyrimidine dehydrogenase influence outcome following fluoropyrimidine-based chemotherapy for metastatic colorectal cancer. Br J Cancer.

[19] Sadahiro S, Suzuki T, Tanaka A, Okada K, Nagase H, Uchida J (2012). Association of right-sided tumors with high thymidine phosphorylase gene expression levels and the response to oral uracil and tegafur/leucovorin chemotherapy among patients with colorectal cancer. Cancer Chemother Pharmacol.

[20] Seithel A, Karlsson J, Hilgendorf C, Björquist A, Ungell AL (2006). Variability in mRNA expression of ABC- and SLC-transporters in human intestinal cells: comparison between human segments and Caco-2 cells. Eur J Pharm Sci.

[21] Odin E, Wettergren Y, Nilsson S (2003). Altered gene expression of folate enzymes in adjacent mucosa is associated with outcome of colorectal cancer patients. Clin Cancer Res.

[22] Hlavata I, Mohelnikova-Duchonova B, Vaclavikova R (2012). The role of ABC transporters in progression and clinical outcome of colorectal cancer. Mutagenesis.

